# Effects of thienopyridine class antiplatelets on bleeding outcomes following robot-assisted radical prostatectomy

**DOI:** 10.1038/s41598-024-56570-9

**Published:** 2024-03-11

**Authors:** Masashi Kubota, Mutsushi Kawakita, Satomi Yoshida, Hiroko Kimura, Takayuki Sumiyoshi, Toshinari Yamasaki, Kazuhiro Okumura, Koji Yoshimura, Yoshiyuki Matsui, Kyohei Sugiyama, Hiroshi Okuno, Takehiko Segawa, Yosuke Shimizu, Noriyuki Ito, Hiroyuki Onishi, Satoshi Ishitoya, Takeshi Soda, Toru Yoshida, Yuichi Uemura, Hiroshi Iwamura, Kazutoshi Okubo, Ryosuke Suzuki, Shigeki Fukuzawa, Toshiya Akao, Ryoma Kurahashi, Kimihiro Shimatani, Yuya Sekine, Hiromitsu Negoro, Shusuke Akamatsu, Toshiyuki Kamoto, Osamu Ogawa, Koji Kawakami, Takashi Kobayashi, Takayuki Goto

**Affiliations:** 1 Department of Urology, Kyoto University Graduate School of Medicine, Kyoto, Japan; 2 Department of Urology, Kobe City Medical Center General Hospital, Kobe, Hyogo Japan; 3 Department of Pharmacoepidemiology, Graduate School of Medicine and Public Health, Kyoto University, Kyoto, Japan; 4 Department of Urology, Tenri Yorozu Hospital, Nara, Japan; 5 Department of Urology, Shizuoka General Hospital, Shizuoka, Japan; 6 Department of Urology, National Cancer Center Hospital, Tokyo, Japan; 7 Department of Urology, Kurashiki Central Hospital, Okayama, Japan; 8 Department of Urology, National Hospital Organization Kyoto Medical Center, Kyoto, Japan; 9 Department of Urology, Kyoto City Hospital, Kyoto, Japan; 10 Department of Urology, Kobe City Nishi-Kobe Medical Center, Kobe, Hyogo Japan; 11 Department of Urology, Japanese Red Cross Wakayama Medical Center, Wakayama, Japan; 12 Department of Urology, Japanese Red Cross Osaka Hospital, Osaka, Japan; 13 Department of Urology, Japanese Red Cross Otsu Hospital, Otsu, Shiga Japan; 14 Department of Urology, Kitano Hospital, Osaka, Japan; 15 Department of Urology, Shiga General Hospital, Moriyama, Shiga Japan; 16 Department of Urology, Toyooka Hospital, Toyooka, Hyogo Japan; 17 Department of Urology, Himeji Medical Center, Himeji, Hyogo Japan; 18 Department of Urology, Kyoto Katsura Hospital, Kyoto, Japan; 19 Department of Urology, Numazu City Hospital, Shizuoka, Japan; 20 Department of Urology, Shimada General Medical Center, Shizuoka, Japan; 21 Department of Urology, Rakuwakai Otowa Hospital, Kyoto, Japan; 22 Department of Urology, Faculty of Life Sciences, Kumamoto University, Kumamoto, Japan; 23 Department of Urology, Hyogo Medical University, Nishinomiya, Hyogo Japan; 24 Department of Urology, Akita University Graduate School of Medicine, Akita, Japan; 25 Department of Urology, University of Tsukuba, Tsukuba, Ibaraki Japan; 26 Department of Urology, Nagoya University, Nagoya, Aichi Japan; 27 Department of Urology, Miyazaki University, Miyazaki, Japan

**Keywords:** Bleeding, Prostatectomy, Antiplatelet agents, Aspirin, Clopidogrel, Prostate cancer, Outcomes research

## Abstract

This study aimed to assess the effects of thienopyridine-class antiplatelet agents (including ticlopidine, clopidogrel, and prasugrel) on bleeding complications in patients who underwent robot-assisted radical prostatectomy. This cohort study used a database for robot-assisted radical prostatectomy at 23 tertiary centers nationwide between 2011 and 2022. Patients who received thienopyridines (thienopyridine group) were compared with those who received aspirin monotherapy (aspirin group). The primary outcome was the incidence of bleeding complications. High-grade complications were defined as Clavien–Dindo grade III or higher. The risks of these outcomes were evaluated using inverse probability of treatment weighted regression models. The study results demonstrated that thienopyridine therapy was associated with a higher risk of overall bleeding complications (OR: 3.62, 95%CI 1.54–8.49). The increased risks of the thienopyridine group were detected for low-grade bleeding complications (OR: 3.20, 95%CI 1.23–8.30) but not for high-grade bleeding complications (OR: 5.23, 95%CI 0.78–34.9). The increased risk of bleeding complications was not observed when thienopyridine was discontinued (OR: 2.52, 95%CI 0.83–7.70); however, it became apparent when it was continued perioperatively (OR: 4.35, 95%CI 1.14–16.61). In conclusion, thienopyridine increased the incidence of bleeding complications, particularly low-grade bleeding complications, following robot-assisted radical prostatectomy. These bleeding effects emerged when thienopyridine was continued perioperatively.

## Introduction

Robot-assisted radical prostatectomy (RARP) is the gold standard therapy for localized prostate cancer^1^. Recently, healthier patients with additional thrombotic comorbidities, such as cardiovascular artery disease and cerebral artery stroke, have been increasingly selected for RARP to manage prostate cancer that cannot be addressed using alternative treatment options^2^. Most of these patients are prescribed antiplatelet agents, specifically thienopyridines, which are selective and reversible adenosine diphosphate receptor or P2Y12 inhibitors. Thienopyridines include the first-generation ticlopidine, second-generation clopidogrel, and third-generation prasugrel. These drugs are widely used and are more effective than aspirin monotherapy in preventing the recurrence of thrombotic diseases, such as post-coronary stenting and non-cardiogenic ischemic cerebral vascular diseases^3–6^. However, thienopyridine therapies are associated with increased hemorrhagic adverse events in several indications^7,8^. Urologic surgeries, classified as hemorrhagic procedures, generally require the discontinuation of thienopyridines preoperatively or the implementation of bridging therapies^9–11^. RARP, classified as a hemorrhagic surgery, is associated with less intraoperative bleeding compared with open-approach radical prostatectomy^12^. Recently, the continued perioperative use of low-dose aspirin therapy during RARP is considered safe and has no correlation with increased blood loss or complications^9,13–17^. However, the effect of thienopyridine therapies during RARP has not been established, and there is little information available on the perioperative management of this relatively novel class of antiplatelets. The need to limit indications for RARP in patients receiving thienopyridine therapy owing to potentially fatal hemorrhagic complications requires discussion. Therefore, this study aimed to investigate the effect of thienopyridine administration on bleeding complications during the perioperative period in patients undergoing RARP and to compare its effect with that of aspirin monotherapy.

## Material and methods

### Study cohorts and design

This cohort study utilized data from an approved common database across 23 tertiary care centers nationwide in Japan. In this study, all consecutive patients registered in the database who were diagnosed with clinical T1-4, N0-1, or M0 prostate cancer and underwent RARP between January 2011 and January 2022 were screened for possible retrospective analysis. Details for the study participants can be found in Supplementary information (Supplementary methods). Exclusion criteria involved patients: (1) without a recent history of antiplatelet or anticoagulant therapy, (2) prescribed anticoagulant agents, including warfarin potassium and direct oral anticoagulants, and (3) administered dual antiplatelet therapy (DAPT) without thienopyridine.

The antiplatelets used were thienopyridines (including ticlopidine, clopidogrel, and prasugrel) and acetylsalicylic acid (aspirin). We divided patients who received these antiplatelets daily into the thienopyridine group (antiplatelet therapy including any thienopyridine class) and the aspirin group (monotherapy with acetylsalicylic acid). Subsequently, the surgical and perioperative outcomes of the thienopyridine group were compared with those of the aspirin group.

### Perioperative management of antiplatelets and surgical procedure

Attending surgeons determined whether to discontinue or continue administering antiplatelet agents perioperatively to all patients. Patients with a history of high-risk embolisms, such as an intervention for cardiovascular diseases or stroke, were referred to institutional specialists for multidisciplinary advice on perioperative management. The standard preoperative withdrawal duration for thienopyridines ranges from 7 to 14 days based on the embolism risk of the patient. Patients at thrombotic risk who discontinued their antiplatelet agents perioperatively underwent bridging therapies using heparin derivatives while monitoring the prothrombin time. RARPs were performed using a four-arm da Vinci S, Si, X, Xi^®^–system (Intuitive Surgical, Sunnyvale, CA).

### Study outcomes

The primary outcome was the overall incidence of 90-day bleeding complications that required pharmacological or procedural intervention, including vesical irrigation, catheter insertion for de-clotting, transfusion, secondary surgical, endoscopic, or radiological procedures to treat hemorrhage, and readmission for hemorrhagic complications. High-grade complications were defined as Clavien–Dindo grade^18,19^ III or higher. Secondary outcomes included the incidence of 90-day thrombotic complications that required intervention, perioperative transfusion rate, intraoperative estimated blood loss, hemoglobin deficit, and incidence of 90-day overall high-grade complications.

### Statistical analysis

Inverse probability of treatment weighting (IPTW) using propensity scores was conducted to balance differences in baseline characteristics between the thienopyridine and aspirin groups. Propensity scores were calculated using logistic regression analysis, with the thienopyridine group as the dependent variable and age (years), American Society of Anesthesiologists physical status [ASA PS] (grades 1–2 vs. 3 and more), body mass index [BMI] (< 25 vs. ≥ 25 kg/m^2^), chronic kidney disease [CKD] grades (Kidney Disease Improving Global Outcomes [KDIGO] classification^20^) (grades 1–2 vs. 3a and more), National Comprehensive Cancer Network [NCCN] risk classification (low to intermediate vs. high-risk group), lymph node dissection (none or limited vs. extended or more), neurovascular bundle preservation (negative vs. positive), preoperative hormonal therapy (negative vs. positive), and perioperative continuation of antiplatelets (continuation vs. discontinuation) as independent variables. The estimated IPTWs were truncated at the 99th percentiles. The balance between covariates in the weighted groups was also assessed using the standardized difference^21^ and plotting their distribution with the unweighted data. Weighted logistic and linear regression analyses were performed using thienopyridine and aspirin groups as covariates and bleeding complications and other outcomes as outcome variables. Subgroup analyses were conducted to investigate the effect of the continuation or discontinuation of antiplatelet therapy in the thienopyridine and aspirin groups during RARP. Patients in the thienopyridine and aspirin groups were divided into perioperative continuation or discontinuation cohorts based on the management with thienopyridines or aspirin, respectively. The difference in backgrounds, bleeding risk and other outcomes between the two groups was revaluated in the continuation and discontinuation cohorts, respectively.

Statistical analyses were conducted using the JMP ver. 16 software package (SAS Institute, Chicago, IL). The Wilcoxon rank-sum and chi-square tests were used to determine significant differences between groups in the univariate analysis. Statistical significance was set at *p* < 0.05.

### Sensitivity analyses

The several sensitivity analyses focused on addressing concerns about the heterogeneity of patients in the thienopyridine group. We extracted 83 patients who were administered clopidogrel monotherapy from the thienopyridine group and compared them with the aspirin group, weighting using recalculated propensity scores. Additionally, a subgroup analysis was also performed on the cohort, distinguishing between the NCCN high-risk group and others (low- and intermediate-risk groups). This analysis aimed to address concerns regarding the heterogeneity of exposures among surgical procedures based on oncological backgrounds.

### Ethics declarations

All procedures involving human participants were conducted in accordance with the ethical standards of the Institutional Research Committee and the 1964 Helsinki Declaration and its later amendments or comparable ethical standards. The Institutional Review Board of Kyoto University Hospital approved this study and the common study database (R3168). Participants received comprehensive information about the study, including its purpose, procedures, and potential risks. Prior to participation, explicit informed consent was obtained, emphasizing voluntariness and the right to withdraw without penalty. An opt-out mechanism was in place for those who chose not to participate, with clear instructions provided. This procedure was approved by the Institutional Review Board of Kyoto University Hospital, ensuring adherence to ethical standards, participant anonymity, and secure data handling.

## Results

**Figure 1:**
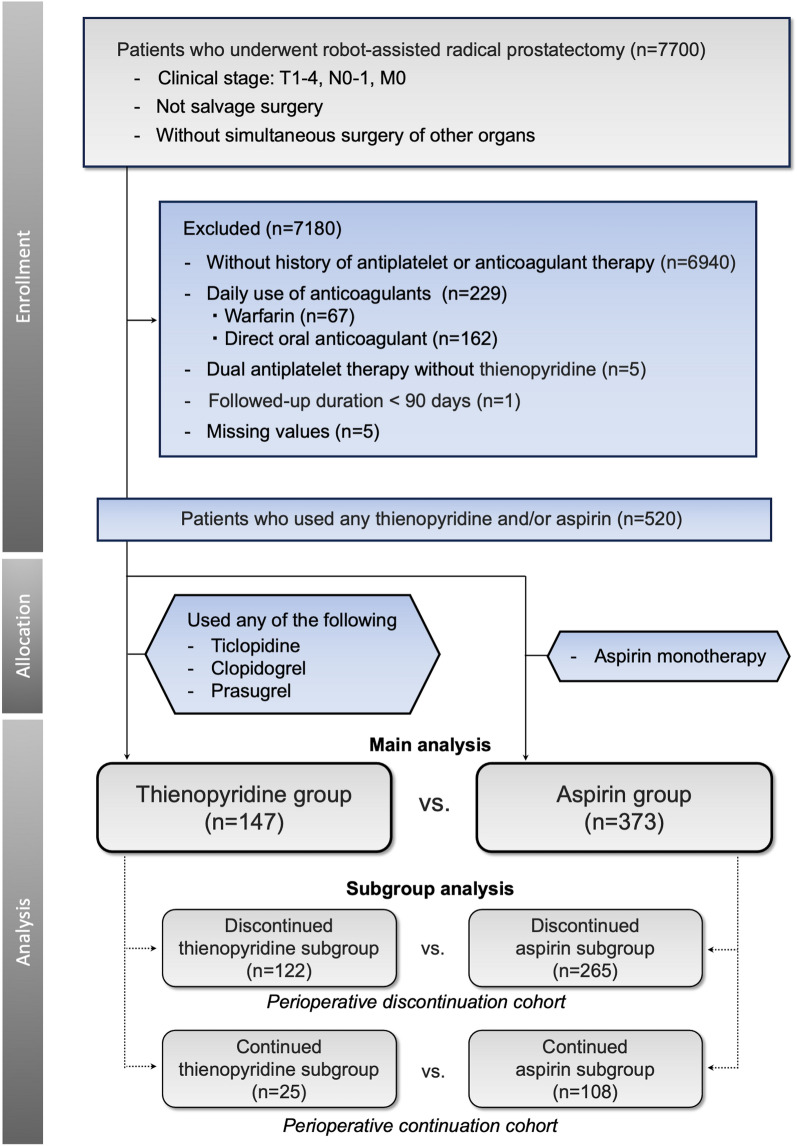
Flow diagram of the study.

Figure 1 depicts a flow diagram of the study. Among the 7700 consecutive patients included in the screening process, 7180 were excluded based on the exclusion criteria (6940 did not use any antiplatelets, 229 used anticoagulants daily, 5 were administered DAPT with aspirin and cilostazol, 1 was lost to follow-up, and 5 had missing data). Consequently, 520 patients, with 147 in the thienopyridine group and 373 in the aspirin group, were included in this study.

Table 1 details the antiplatelet agents used by patients in the thienopyridine and aspirin groups. Among the 147 patients in the thienopyridine group, 11 (7%), 126 (86%), and 10 (7%) received ticlopidine, clopidogrel, and prasugrel, respectively. Additionally, 52 (35%) were administrated DAPT combined with aspirin or cilostazol. The aspirin group comprised 373 patients who received aspirin monotherapy. In the thienopyridine and aspirin groups, antiplatelet agents were mainly used for the treatment or secondary prevention of coronary artery disease and cerebral infarction.

**Table 1:** Details of administered antiplatelets in the thienopyridine and aspirin groups.

Parameter	Overall	Thienopyridine group	Aspirin group
Patients, no	520	147	373
Administered antiplatelets, n (%)			
Thienopyridine	147 (28)	147 (100)	–
Ticlopidine	11 (2)	11 (7)	–
Clopidogrel	126 (24)	126 (86)	–
25 mg	28 (5)	28 (19)	–
50 mg	14 (3)	14 (10)	–
75 mg	84 (16)	84 (57)	–
Prasugrel	10 (2)	10 (7)	–
Aspirin	422 (81)	49 (33)	373 (100)
100 mg	410 (79)	47 (32)	363 (97)
200 mg, or more	12 (2)	2 (1)	10 (3)
Cilostazol 100 mg	3 (0.6)	3 (2)	–
DAPT	52 (10)	52 (35)	–
History, n (%)			
Coronary artery disease	231 (44)	65 (44)	166 (45)
Cerebral infarction	118 (23)	44 (30)	74 (20)
Carotid stenosis	41 (8)	15 (10)	26 (7)
Arrhythmia	22 (4)	4 (3)	18 (5)
Cardiac valve surgery	4 (0.8)	1 (0.7)	3 (0.8)
Primary prevention	23 (4)	2 (1)	21 (6)
Others	111 (21)	20 (14)	91 (24)

*DAPT* dual antiplatelet therapy

**Figure 2:**
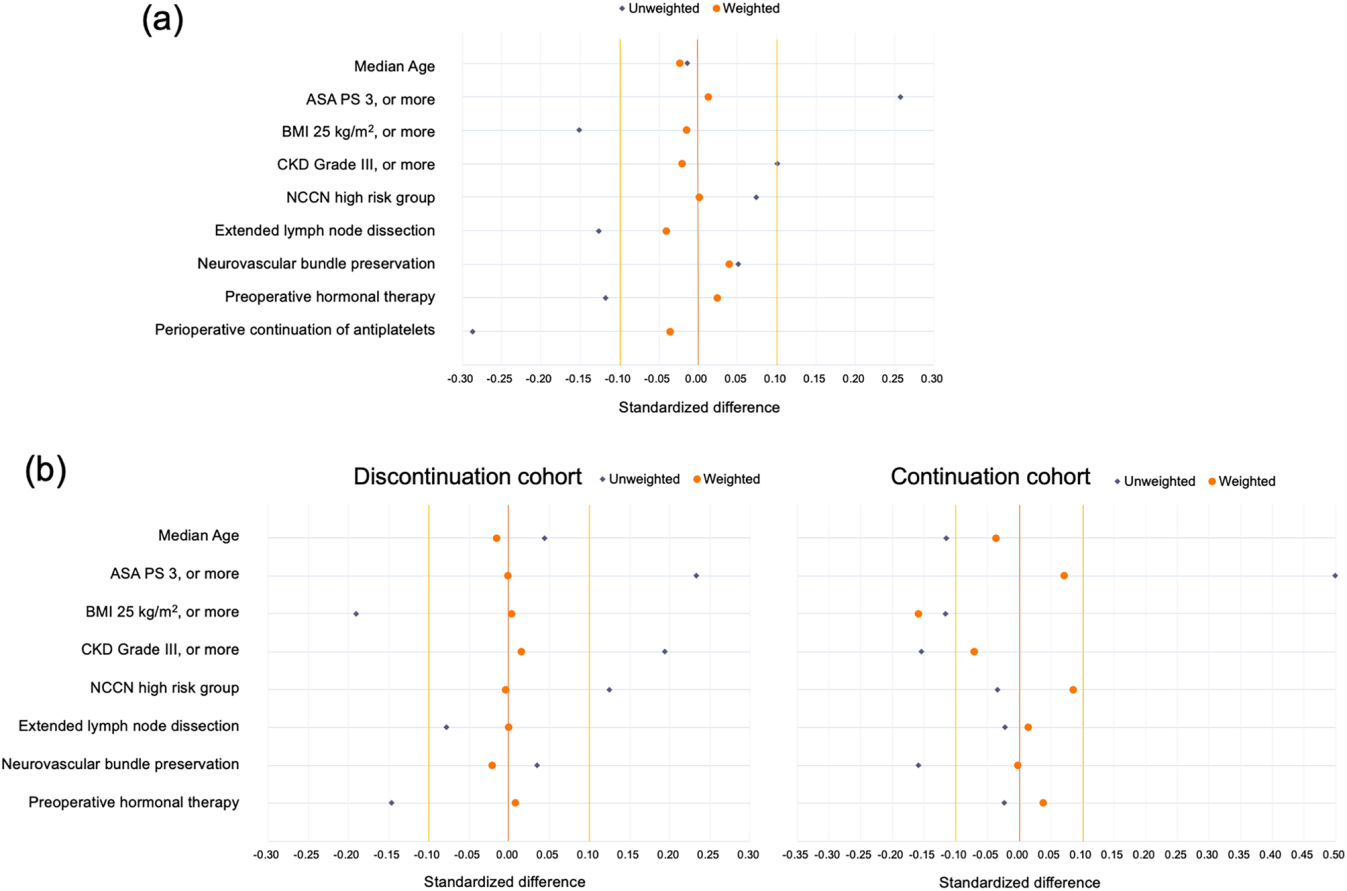
Standardized differences plot before and after IPTW in (**a**) main analysis and (**b**) subgroup analysis of the study.

Table 2 and Fig. 2 presents the patient backgrounds for this study. Before conducting IPTW, patients in the thienopyridine group had higher ASA PS scores of 3 or more and KDIGO CKD grade 3 or more than those in the aspirin group. In contrast, patients in the aspirin group had higher body mass index ≥ 25 kg/m^2^, extended lymph node dissection, preoperative hormonal therapy, and perioperative continuation of antiplatelet therapy compared with those in the thienopyridine group. After IPTW adjustment, the standardized differences for all baseline characteristics were within 10% between the two groups.

**Table 2:** Patient characteristics before and after IPTW compared between the thienopyridine and aspirin groups.

Parameter	Total	Unweighted study cohort				Weighted study cohort			
		Thienopyridine group	vs	Aspirin group	SD	Thienopyridine group	vs	Aspirin group	SD
**Patients, no**	**520**	**147**		**373**					
Median age, years (IQR)	71 (68–74)	71 (67–74)		71 (68–74)	− 0.014	71 (67–75)		71 (68–74)	− 0.023
ASA PS, n (%)									
1	63 (12)	17 (11)		46 (12)	**0.257**	13%		12%	0.013
2	358 (69)	91 (62)		267 (72)		67%		69%	
3, or more	99 (19)	39 (27)		60 (16)		20%		19%	
BMI ≧ 25 kg/m^2^, n (%)	204 (39)	50 (34)		154 (41)	**− 0.151**	38%		39%	− 0.015
KDIGO CKD grade (eGFR), n (%)									
Grade 1, or 2 (60 mL/min/1.73 m^2^, or more)	333 (64)	89 (61)		244 (65)	**0.101**	65%		64%	− 0.020
Grade 3a, or 3b (30–59 mL/min/1.73 m^2^)	172 (33)	51 (35)		121 (32)		32%		33%	
Grade 4, or 5 (29 mL/min/1.73 m^2^, or less)	15 (2.9)	7 (4.7)		8 (2.1)		3.4%		3.1%	
NCCN risk classification group, n (%)									
Low	15 (2.9)	4 (2.9)		11 (3.0)	0.074	2.6%		3.0%	0.002
Intermediate	257 (49)	69 (47)		188 (40)		49%		49%	
High, or more	248 (48)	74 (50)		174 (47)		48%		48%	
Pelvic lymph node dissection, n (%)									
None	269 (51)	75 (51)		194 (52)	**− 0.126**	52%		53%	− 0.040
Limited	144 (28)	47 (32)		97 (26)		29%		27%	
Extended	107 (21)	25 (17)		82 (22)		19%		20%	
Neurovascular bundle preservation, n (%)	154 (30)	46 (31)		108 (29)	0.051	29%		29%	0.040
Preoperative hormonal therapy, n (%)	132 (25)	32 (22)		100 (27)	**− 0.118**	27%		26%	0.024
Perioperative continuation of antiplatelets, n (%)	133 (26)	25 (17)		108 (29)	**− 0.287**	24%		26%	− 0.036

ASA PS, American Society of Anesthesiologists physical status classification; BMI, body mass index; CKD, chronic kidney disease; eGFR, estimated glomerular filtration rate; IPTW, inverse probability of treatment weighting; IQR, interquartile range; KDIGO, kidney disease improving global outcomes; NCCN, National Comprehensive Cancer Network; SD, Standardized difference.

Significant values are in bold.

Supplementary Table S1 provides the details of bleeding and other study outcomes. The bleeding sites of all patients were investigated and were limited to the pelvis or lower urinary tract. Only one patient in each group developed hemorrhagic shock from postoperative bleeding from the internal iliac artery and required catheter embolization. None of the patients died from perioperative bleeding; however, one patient in each group, who had discontinued antiplatelet therapy, died because of the rapid deterioration of their underlying myocardial infarction and stroke conditions.

Table 3 shows the results of the logistic and linear regression analyses that evaluated the association between the thienopyridine and aspirin groups regarding bleeding complications and other outcomes. The unweighted analysis revealed no significant differences in the risk of overall bleeding complications (odds ratio [OR] = 3.13 [95% confidence interval (CI), 0.94–10.43], *p* = 0.063) and all other outcomes. However, the IPTW-adjusted analysis revealed significant differences between the thienopyridine and aspirin groups in the incidence risk of overall bleeding complications (OR = 3.62 [95% CI 1.54–8.49], *p* = 0.003), transfusion rate (OR = 6.35 [95% CI 1.75–23.01], *p* = 0.005), and readmission rate (OR = 2.96 [95% CI 1.34–6.54], *p* = 0.007). Among the risks of bleeding complications, a significant difference was observed in the risk of low-grade (C–D grade II or less) bleeding complications (OR = 3.20 [95% CI 1.23–8.30], *p* = 0.017), whereas no significant difference was detected for high-grade (C–D grade III or more) bleeding complications (OR = 5.23 [95% CI 0.78–34.90], *p* = 0.088). The risk of thrombotic complications (OR = 0.27 [95% CI 0.05–1.50], *p* = 0.13) did not differ significantly between the two groups.

**Table 3:** Unweighted and weighted regression models analyzing associations between the study outcomes and the thienopyridine group compared with the aspirin group.

Parameters	Thienopyridine group vs. Aspirin group (Ref.)					
	Unweighted analysis			IPTW analysis		
Binary outcomes	Odds ratio	95% CI	*P*-value	Odds ratio	95% CI	*P*-value
Bleeding complications	3.13	0.94–10.43	0.063	3.62	1.54–8.49	0.003
Low grade (C–D grade II or less)	3.24	0.86–12.27	0.082	3.20	1.23–8.30	0.017
High grade (C–D grade III or more)	2.55	0.16–41.01	0.51	5.23	0.78–34.90	0.088
Transfusion	3.86	0.64–23.37	0.14	6.35	1.75–23.01	0.005
Hemorrhagic shock	2.55	0.16–41.01	0.51	5.23	0.78–34.90	0.088
Thrombotic complication	0.68	0.070–5.70	0.68	0.27	0.05–1.50	0.13
Overall high-grade complications	1.50	0.58–3.90	0.40	1.31	0.70–2.45	0.40
Readmission	2.60	0.83–8.20	0.10	2.96	1.34–6.54	0.007

Continuous outcomes	Estimate	95% CI	*P*-value	Estimate	95% CI	*P*-value
Operation time, min	0.33	− 7.60 to 8.26	0.93	− 2.30	− 9.57 to 4.97	0.53
Estimated blood loss, mL	3.04	− 19.13 to 25.22	0.79	− 9.26	− 32.50 to 13.99	0.43
Hemoglobin deficit, median, mg/dL	0.001	− 0.10 to 0.11	0.98	− 0.004	− 0.10 to 0.09	0.94

C–D grade, Clavien–Dindo grade; CI, Confidence interval; IPTW, inverse probability of treatment weighting.

Table 4 and Supplementary Table S2 present the results of the subgroup analysis of the study. Even after IPTW was applied, the BMI value in the continuation cohort remained higher in the aspirin subgroup than in the thienopyridine subgroup. In the cohort of perioperative discontinuation of antiplatelets, no significant differences were observed in the incidence risk of overall bleeding complications (OR = 2.52 [95% CI 0.83–7.70], *p* = 0.10) and other outcomes by the IPTW-regression analyses. However, in the cohort of perioperative continuation of antiplatelets, significant differences were observed in the IPTW-regression analyses between the continuation thienopyridine and aspirin subgroups in terms of the risk of overall bleeding complications (OR = 4.35 [95% CI 1.14–16.61], *p* = 0.031), transfusion rate (OR = 8.66 [95% CI 1.48–50.73], *p* = 0.017), and readmission rate (OR = 5.04 [95% CI 1.18–21.47], *p* = 0.029).

**Table 4:** Subgroup analyses: IPTW-regression models analyzed associations between the outcomes and the thienopyridine group compared with the aspirin group in the perioperative discontinuation (left) and continuation (right) of antiplatelet cohorts.

Parameters	Thienopyridine subgroup vs. Aspirin subgroup (Ref.)					
	Perioperative discontinuation cohort			Perioperative continuation cohort		
Binary outcomes	Odds ratio	95% CI	*P*-value	Odds ratio	95% CI	*P*-value
Bleeding complications	2.52	0.83–7.70	0.10	4.35	1.14–16.61	0.031
Low grade (C–D grade II or less)	2.52	0.83–7.70	0.10	4.45	0.68–29.19	0.12
High grade (C–D grade III or more)	NA	NA	NA	3.97	0.61–25.85	0.15
Transfusion	2.42	0.35–17.00	0.37	8.66	1.48–50.73	0.017
Hemorrhagic shock	NA	NA	NA	3.97	0.61–25.85	0.15
Thrombotic complication	0.42	0.064–2.70	0.36	NA	NA	NA
Readmission	2.31	0.89–6.02	0.087	5.04	1.18–21.47	0.029

Continuous outcomes	Estimate	95% CI	*P*-value	Estimate	95% CI	*P*-value
Operation time, min	− 3.80	− 12.09 to 4.48	0.37	− 1.53	− 16.07 to 13.02	0.84
Estimated blood loss, mL	− 8.15	− 30.08 to 13.76	0.46	− 13.44	− 75.20 to 48.32	0.67
Hemoglobin deficit, median, mg/dL	− 0.020	− 0.13 to 0.09	0.72	0.020	− 0.16 to 0.20	0.83

C–D grade, Clavien–Dindo grade; CI, Confidence interval; IPTW, inverse probability of treatment weighting; NA, not available data for zero event.

In the sensitivity analyses, significant differences were also observed between the clopidogrel monotherapy and aspirin groups in the risk of overall bleeding complications (OR = 6.64 [95% CI 2.78–15.9], *p* < 0.001) and transfusion rate (OR = 14.3 [95% CI 3.75–54.53], *p* < 0.001) (Supplementary Table S3). In the subgroup analysis based on NCCN risk groups, the difference in bleeding risk between the thienopyridine and aspirin groups was also replicated in the cohorts of NCCN High-risk (OR = 4.66 [95% CI 1.43–15.22], *p* = 0.011) and NCCN Low and Intermediate risk (OR = 3.40 [95% CI 1.05–11.01], *p* = 0.041), respectively (Supplementary Table S4).

## Discussion

This study compared the effect of thienopyridine and aspirin on post-RARP bleeding complications. We found that patients receiving thienopyridine had a higher risk of low-grade bleeding complications requiring primarily conservative management than patients who received aspirin.

To our knowledge, this is the first study to investigate the hemorrhagic characteristics following RARP in patients administered thienopyridine compared with those administered aspirin monotherapy. A recent meta-analysis examining the effect of aspirin, clopidogrel, and DAPT in patients undergoing non-cardiac surgery revealed an increased need for blood transfusion with increased platelet inhibition. However, no difference was observed in the need for secondary procedures for bleeding following surgery^22^. In the urologic robot surgery field, clopidogrel increases the risk of bleeding in robot-assisted partial nephrectomy, whereas perioperative continuation of aspirin is considered safe^23^. The results of our study, which focused on RARP, were consistent with those of prior studies, indicating that clopidogrel increased the risk of non-interventional bleeding complications, including the need for transfusion. Our study provided a more detailed characterization of the properties of thienopyridines by focusing on bleeding complications specific to radical prostatectomy, such as bladder tamponade and pelvic oozing. The results of this study showed that patients who continue thienopyridines perioperatively need to be managed for postoperative bleeding following RARP, mainly by bedside management, such as careful postoperative observation, urinary tract management, and transfusions.

Moreover, patients who received aspirin monotherapy were designated as the control group in our study. Most of the previous studies that investigated the effect of antiplatelet agents in urological surgery compared their outcomes with those of healthy patients who were not administered such blood thinners. However, this comparison design included indication bias as most patients with a history of vascular diseases requiring blood thinners already had other bleeding risks due to comorbidities, such as hypertension, CKD, and other conditions related to vascular vulnerability. Our study design played an important role in decreasing such bias by comparing patients who were administered thienopyridines with those from similar disease backgrounds. One of the possible mechanisms explaining the difference in postoperative bleeding between thienopyridines and aspirin is the higher prevalence of CYP2C19 single nucleotide polymorphisms in East Asians^24^. In association with these genetic polymorphisms, it has been demonstrated that the use of thienopyridines may have no effect in reducing the ischemic risk while significantly increasing the bleeding risk in East Asians^25,26^. The difference in bleeding risk based on ethnic backgrounds specific to thienopyridines, which is not observed in aspirin, has the potential to emphasize the results of our study, limited to the Japanese population cohort.

Despite the result of the study, the safety of perioperative discontinuation of thienopyridines during RARP remains controversial. In the subgroup analysis of the study, two (0.5%) out of 387 patients in the perioperative discontinuation of antiplatelet cohort died from thrombotic complications, while none of the patients in the perioperative continuation of antiplatelet cohort died from perioperative bleeding. Due to the absence of thrombotic complications in the perioperative continuation of antiplatelet cohort, it was not feasible to analyze the risk evaluation between bleeding and embolism. However, our results may generate the hypothesis that it seems one of the reasonable choices to continue thienopyridines perioperatively and prepare for watchful monitoring of bleeding to avoid lethal complications following RARP in thienopyridine users.

Our study has some limitations. This retrospective cohort study included a small population in the thienopyridine group. No significant difference was discovered in the incidence of bleeding complications requiring secondary procedures between the thienopyridine and other groups. However, a larger study with a calculated sample size is required to further clarify the non-inferiority of outcomes in the thienopyridine group. Moreover, this observational study was subject to selection bias. As this study only included patients who chose surgical treatment, patients at higher risk of complications may have chosen alternative treatment options. Due to the cohort being derived from surgical records, patients who were unable to undergo RARP because of the preoperative onset of thrombosis resulting from the discontinuation of thienopyridine or aspirin were excluded from the study. The study has not accounted for certain confounding factors, such as the heterogeneity of surgical skills among surgeons and patient-related factors associated with the surgical difficulties of RARP, including a high-volume prostate and a small pelvis. In conclusion, this study revealed that administering thienopyridine-class antiplatelet therapies may be associated with an increased risk of bleeding complications, transfusions, and readmission, particularly low-grade bleeding complications that did not require secondary procedures, in patients who underwent RARP. These bleeding effects of thienopyridine were not observed when it was discontinued perioperatively; however, they emerged when it was continued during RARP.

### Supplementary Information

Supplementary Tables.

## Data Availability

The data sets of the study are not publicly available as they contain information that could compromise the privacy of research participants but are available from the corresponding author on reasonable request.

## Abbreviations

ASA PS: American Society of Anesthesiologists physical status
CKD: Chronic kidney disease
DAPT: Dual antiplatelet therapy
IPTW: Inverse probability of treatment weighting
KDIGO: Kidney Disease Improving Global Outcomes
RARP: Robot-assisted radical prostatectomy
