# Supplementary material for: Effects of thienopyridine class antiplatelets on bleeding outcomes following robot-assisted radical prostatectomy
Source: Sci Rep. 2024 Mar 11;14:5847. doi: 10.1038/s41598-024-56570-9 (PMC10925592; doi:10.1038/s41598-024-56570-9)
Supplement: Supplementary file 1 — Supplementary Tables. [file 41598_2024_56570_MOESM1_ESM.docx]

**Supplementary information**

**Supplementary methods.** Participants of the study database.

In the study, the data of included patients were collected at Kyoto University Graduate School of Medicine, Kyoto, Japan; Kobe City Medical Center General Hospital, Hyogo, Japan; Kumamoto University, Kumamoto, Japan; Tsukuba University, Ibaraki, Japan; Miyazaki University, Miyazaki, Japan; Akita University Graduate School of Medicine, Akita, Japan; Hyogo Medical University, Hyogo, Japan; National Cancer Center Hospital, Tokyo, Japan; Kobe City Nishi-Kobe Medical Center, Hyogo, Japan; Tenri Yorozu Hospital, Nara, Japan; Kurashiki Central Hospital, Okayama, Japan; Shizuoka General Hospital, Shizuoka, Japan; Kitano Hospital, Osaka, Japan; Kyoto Medical Center, Kyoto, Japan; Japanese Red Cross Osaka Hospital, Osaka, Japan; Japanese Red Cross Otsu Hospital, Shiga, Japan; Kyoto City Hospital, Kyoto, Japan; Shiga General Hospital, Moriyama, Japan; Toyooka Hospital, Hyogo, Japan; Himeji Medical Center, Hyogo, Japan; Numazu City Hospital, Shizuoka, Japan; Shimada General Medical Center, Shizuoka, Japan; and Rakuwakai Otowa Hospital, Kyoto, Japan.

| **Supplementary Table S1.** The details of the incidences of bleeding complications and other surgical outcomes of the two groups. | | | | | | | |
| --- | --- | --- | --- | --- | --- | --- | --- |
|  |  |  |  | | | | |
| Parameter | Total |  | Thienopyridine group | vs. | Aspirin group |  | p-value |
| **Patients, no.** | **520** |  | **147** |  | **373** |  |  |
| Bleeding complication, n (%) | 11 (2.1) |  | 6 (4.1) |  | 5 (1.3) |  | 0.058 |
| Low grade (C–D grade II or less), n (%) | 9 (1.7) |  | 5 (3.4) |  | 4 (1.1) |  | 0.077 |
| High grade (C–D grade III or more), n (%) | 2 (0.4) |  | 1 (0.7) |  | 1 (0.3) |  | 0.49 |
| Hemorrhagic shock, n (%) | 2 (0.4) |  | 1 (0.7) |  | 1 (0.3) |  | 0.49 |
| Hemorrhagic death, n (%) | 0 (0) |  | 0 (0) |  | 0 (0) |  | 1.00 |
| Transfusion, n (%) | 5 (0.9) |  | 3 (2.0) |  | 2 (0.5) |  | 0.14 |
| Bleeding sites, n (%) |  |  |  |  |  |  |  |
| Pelvis | 7 (1.3) |  | 4 (1.4) |  | 3 (0.8) |  | 0.10 |
| Lower urinary tracts | 4 (0.8) |  | 2 (2.7) |  | 2 (0.5) |  | 0.32 |
| Thrombotic complication, n (%) | 5 (0.9) |  | 1 (0.7) |  | 4 (1.1) |  | 0.81 |
| Low grade (C–D grade II or less), n (%) | 2 (0.4) |  | 0 (0) |  | 2 (0.5) |  | 0.51 |
| High grade (C–D grade III or more), n (%) | 3 (0.6) |  | 1 (0.7) |  | 2 (0.5) |  | 0.63 |
| Thrombotic death, n (%) | 1 (0.2) |  | 1 (0.7) |  | 1 (0.3) |  | 0.49 |
| Overall high-grade complications, n (%) | 19 (3.7) |  | 7 (4.8) |  | 12 (3.2) |  | 0.27 |
| Hospital stay, median, POD (IQR) | 8 (7-10) |  | 9 (7-10) |  | 8 (7-10) |  | 0.66 |
| Readmission, n (%) | 12 (2.3) |  | 6 (4.1) |  | 6 (1.6) |  | 0.090 |
| Mortalities, n (%) | 1 (0.2) |  | 1 (0.7) |  | 1 (0.3) |  | 0.49 |
|  |  |  |  |  |  |  |  |
| Operation time, median, min (IQR) | 244 (198–308) |  | 243 (195–307) |  | 244 (200–308) |  | 0.74 |
| Estimated blood loss, median, mL (IQR) | 100 (24–250) |  | 89 (10–220) |  | 100 (30–262) |  | 0.18 |
| Hemoglobin deficit, median, mg/dL (IQR) | 1.7 (1.0–2.5) |  | 1.8 (1.0–2.5) |  | 1.7 (1.0–2.4) |  | 0.84 |
|  |  |  |  |  |  |  |  |
| ^C–D grade, Clavien–Dindo grade; IQR, interquartile range; POD, postoperative day.^ | | | | | | | |

| **Supplementary Table S2.** Patient characteristics of subgroup analyses before and after IPTW compared between the thienopyridine and aspirin subgroups in (a) perioperative discontinuation of the antiplatelets cohort and (b) perioperative continuation of the antiplatelets cohort. | | | | | | | | | | | | | | | | | | | | |  |  |  |  |
| --- | --- | --- | --- | --- | --- | --- | --- | --- | --- | --- | --- | --- | --- | --- | --- | --- | --- | --- | --- | --- | --- | --- | --- | --- |
| (a) |  | |  | |  | |  | |  | |  |  | | |  | |  | |  | |  |  |  |  |
| ***Perioperative discontinuation cohort*** |  | | ***Unweighted study cohort*** | | | | | | | |  | ***Weighted study cohort*** | | | | | | | | |  |  |  |  |
| Parameter |  | | Thienopyridine subgroup | | vs. | | Aspirin subgroup | | SD | |  | Thienopyridine subgroup | | | vs. | | Aspirin subgroup | | SD | |  |  |  |  |
| **Patients, no.** |  | | **122** | |  | | **265** | |  | |  |  | | |  | |  | |  | |  |  |  |  |
| Median age, years (IQR) |  | | 71 (67–75) | |  | | 71 (68–74) | | 0.044 | |  | 71 (67–75) | | |  | | 71 (68–74) | | -0.016 | |  |  |  |  |
| ASA PS, n (%) |  | |  | |  | |  | |  | |  |  | | |  | |  | |  | |  |  |  |  |
| 1 |  | | 13 (11) | |  | | 32 (12) | | 0.233 | |  | 11% | | |  | | 12% | | -0.002 | |  |  |  |  |
| 2 |  | | 80 (66) | |  | | 196 (74) | |  | |  | 72% | | |  | | 71% | |  | |  |  |  |  |
| 3, or more |  | | 28 (23) | |  | | 37 (14) | |  | |  | 17% | | |  | | 17% | |  | |  |  |  |  |
| BMI ≧ 25 kg/m2, n (%) |  | | 43 (35) | |  | | 118 (45) | | -0.190 | |  | 42% | | |  | | 42% | | 0.003 | |  |  |  |  |
| KDIGO CKD grade (eGFR), n (%) |  | |  | |  | |  | |  | |  |  | | |  | |  | |  | |  |  |  |  |
| Grade 1, or 2 (60 mL/min/1.73 m^2^, or more) |  | | 73 (60) | |  | | 183 (69) | | 0.194 | |  | 65% | | |  | | 66% | | 0.015 | |  |  |  |  |
| Grade 3a, or 3b (30–59 mL/min/1.73 m^2^) |  | | 45 (37) | |  | | 78 (29) | |  | |  | 33% | | |  | | 32% | |  | |  |  |  |  |
| Grade 4, or 5 (29 mL/min/1.73 m^2^, or less) |  | | 4 (3.3) | |  | | 4 (1.5) | |  | |  | 2.2% | | |  | | 2.1% | |  | |  |  |  |  |
| NCCN risk classification group, n (%) |  | |  | |  | |  | |  | |  |  | | |  | |  | |  | |  |  |  |  |
| Low |  | | 2 (1.6) | |  | | 5 (1.9) | | 0.125 | |  | 1.0% | | |  | | 1.9% | | -0.004 | |  |  |  |  |
| Intermediate |  | | 59 (48) | |  | | 144 (54) | |  | |  | 54% | | |  | | 53% | |  | |  |  |  |  |
| High, or more |  | | 61 (50) | |  | | 116 (44) | |  | |  | 45% | | |  | | 45% | |  | |  |  |  |  |
| Pelvic lymph node dissection, n (%) |  | | 16 (13) | |  | | 42 (16) | |  | |  | 15% | | |  | | 15% | |  | |  |  |  |  |
| None |  | | 61 (50) | |  | | 137 (52) | | -0.078 | |  | 50% | | |  | | 52% | | -0.001 | |  |  |  |  |
| Limited |  | | 45 (37) | |  | | 86 (32) | |  | |  | 35% | | |  | | 33% | |  | |  |  |  |  |
| Extended |  | | 16 (13) | |  | | 42 (16) | |  | |  | 15% | | |  | | 15% | |  | |  |  |  |  |
| Neurovascular bundle preservation, n (%) |  | | 43 (35) | |  | | 89 (34) | | 0.035 | |  | 33% | | |  | | 34% | | -0.021 | |  |  |  |  |
| Preoperative hormonal therapy, n (%) |  | | 26 (21) | |  | | 73 (28) | | -0.146 | |  | 26% | | |  | | 26% | | 0.008 | |  |  |  |  |
| (b) |  | |  | |  | |  | |  | |  |  | | |  | |  | |  | |  |  |  |  |
| ***Perioperative continuation cohort*** |  | | ***Unweighted study cohort*** | | | | | | | |  | ***Weighted study cohort*** | | | | | | | | |  |  |  |  |
| Parameter |  | | Thienopyridine subgroup | | vs. | | Aspirin subgroup | | SD | |  | Thienopyridine subgroup | | | vs. | | Aspirin subgroup | | SD | |  |  |  |  |
| **Patients, no.** |  | | **25** | |  | | **108** | |  | |  |  | | |  | |  | |  | |  |  |  |  |
| Median age, years (IQR) |  | | 70 (67–75) | |  | | 72 (68–75) | | -0.115 | |  | 70 (67–75) | | |  | | 72 (68–75) | | -0.036 | |  |  |  |  |
| ASA PS, n (%) |  | |  | |  | |  | |  | |  |  | | |  | |  | |  | |  |  |  |  |
| 1 |  | | 4 (16) | |  | | 14 (13) | | 0.499 | |  | 20% | | |  | | 12% | | 0.071 | |  |  |  |  |
| 2 |  | | 10 (40) | |  | | 71 (66) | |  | |  | 50% | | |  | | 62% | |  | |  |  |  |  |
| 3, or more |  | | 11 (44) | |  | | 23 (21) | |  | |  | 30% | | |  | | 26% | |  | |  |  |  |  |
| BMI ≧ 25 kg/m2, n (%) |  | | 7 (28) | |  | | 36 (33) | | -0.116 | |  | 25% | | |  | | 32% | | -0.159 | |  |  |  |  |
| KDIGO CKD grade (eGFR), n (%) |  | |  | |  | |  | |  | |  |  | | |  | |  | |  | |  |  |  |  |
| Grade 1, or 2 (60 mL/min/1.73 m^2^, or more) |  | | 16 (64) | |  | | 61 (56) | | -0.154 | |  | 61% | | |  | | 58% | | -0.071 | |  |  |  |  |
| Grade 3a, or 3b (30–59 mL/min/1.73 m^2^) |  | | 6 (24) | |  | | 43 (40) | |  | |  | 33% | | |  | | 36% | |  | |  |  |  |  |
| Grade 4, or 5 (29 mL/min/1.73 m^2^, or less) |  | | 3 (12) | |  | | 4 (3.7) | |  | |  | 6.1% | | |  | | 5.7% | |  | |  |  |  |  |
| NCCN risk classification group, n (%) |  | |  | |  | |  | |  | |  |  | | |  | |  | |  | |  |  |  |  |
| Low |  | | 2 (8.0) | |  | | 3 (2.8) | | -0.034 | |  | 5.4% | | |  | | 3.1% | | 0.086 | |  |  |  |  |
| Intermediate |  | | 10 (40) | |  | | 47 (43) | |  | |  | 37% | | |  | | 43% | |  | |  |  |  |  |
| High, or more |  | | 13 (52) | |  | | 58 (54) | |  | |  | 58% | | |  | | 54% | |  | |  |  |  |  |
| Pelvic lymph node dissection, n (%) |  | |  | |  | |  | |  | |  |  | | |  | |  | |  | |  |  |  |  |
| None |  | | 14 (56) | |  | | 56 (52) | | -0.022 | |  | 54% | | |  | | 53% | | 0.015 | |  |  |  |  |
| Limited |  | | 2 (8) | |  | | 12 (11) | |  | |  | 8.5% | | |  | | 11% | |  | |  |  |  |  |
| Extended |  | | 9 (36) | |  | | 40 (37) | |  | |  | 37% | | |  | | 36% | |  | |  |  |  |  |
| Neurovascular bundle preservation, n (%) |  | | 3 (12) | |  | | 19 (18) | | -0.158 | |  | 16% | | |  | | 16% | | -0.002 | |  |  |  |  |
| Preoperative hormonal therapy, n (%) |  | | 6 (24) | |  | | 27 (25) | | -0.023 | |  | 27% | | |  | | 25% | | 0.038 | |  |  |  |  |
|  |  | |  | |  | |  | |  | |  |  | | |  | |  | |  | |  |  |  |  |
| ^ASA PS, American Society of Anesthesiologists physical status classification; BMI, body mass index; CKD, chronic kidney disease; eGFR, estimated glomerular filtration rate; IPTW, inverse probability of treatment weighting; IQR, interquartile range; KDIGO, Kidney Disease Improving Global Outcomes; NCCN, National Comprehensive Cancer Network; SD, Standardized difference^ | | | | | | | | | | | | | | | | | | | | |  |  |  |  |
|  |  |  |  |  |  |  |  |  |  |  |  |  |  |  |  |  |  |  |  |  |  | |  |  |
|  | | | | | | | | | | | | | | | | | | | | |  | |  |  |
| **Supplementary Table S3.** Results of the sensitivity analysis (a) Patient characteristics before and after IPTW compared between the clopidogrel monotherapy (n=83) and aspirin groups (n=373). (b) Unweighted and weighted regression models analyzing associations between the study outcomes and the clopidogrel monotherapy group compared with the aspirin group. | | | | | | | | | | | | | | | | | | | | | | |  |  |
| (a) | | |  | |  | |  | |  | |  | | |  |  | |  | |  | |  | |  |  |
|  | | |  | | ***Unweighted study cohort*** | | | | | | | | |  | ***Weighted study cohort*** | | | | | | | |  |  |
| Parameter | | |  | | Clopidogrel monotherapy group | | vs. | | Aspirin group | | SD | | |  | Clopidogrel monotherapy group | | vs. | | Aspirin group | | SD | |  |  |
| **Patients, no.** | | |  | | **83** | |  | | **373** | |  | | |  |  | |  | |  | |  | |  |  |
| Median age, years (IQR) | | |  | | 72 (68–75) | |  | | 71 (68–74) | | 0.097 | | |  | 72 (68–75) | |  | | 71 (68–74) | | -0.030 | |  |  |
| ASA PS, n (%) | | |  | |  | |  | |  | |  | | |  |  | |  | |  | |  | |  |  |
| 1 | | |  | | 13 (16) | |  | | 46 (12) | | 0.172 | | |  | 20% | |  | | 12% | | -0.017 | |  |  |
| 2 | | |  | | 51 (61) | |  | | 267 (72) | |  | | |  | 63% | |  | | 71% | |  | |  |  |
| 3, or more | | |  | | 19 (23) | |  | | 60 (16) | |  | | |  | 17% | |  | | 17% | |  | |  |  |
| BMI ≧ 25 kg/m2, n (%) | | |  | | 24 (29) | |  | | 154 (41) | | -0.261 | | |  | 42% | |  | | 39% | | 0.058 | |  |  |
| KDIGO CKD grade (eGFR), n (%) | | |  | |  | |  | |  | |  | | |  |  | |  | |  | |  | |  |  |
| Grade 1, or 2 (60 mL/min/1.73 m^2^, or more) | | |  | | 50 (60) | |  | | 244 (65) | | 0.107 | | |  | 70% | |  | | 65% | | 0.104 | |  |  |
| Grade 3a, or 3b (30–59 mL/min/1.73 m^2^) | | |  | | 32 (39) | |  | | 121 (32) | |  | | |  | 29% | |  | | 33% | |  | |  |  |
| Grade 4, or 5 (29 mL/min/1.73 m^2^, or less) | | |  | | 1 (1.2) | |  | | 8 (2.1) | |  | | |  | 1.7% | |  | | 2.3% | |  | |  |  |
| NCCN risk classification group, n (%) | | |  | |  | |  | |  | |  | | |  |  | |  | |  | |  | |  |  |
| Low | | |  | | 3 (3.6) | |  | | 11 (3.0) | | 0.072 | | |  | 4.7% | |  | | 2.9% | | -0.072 | |  |  |
| Intermediate | | |  | | 37 (44) | |  | | 188 (50) | |  | | |  | 51% | |  | | 50% | |  | |  |  |
| High, or more | | |  | | 43 (52) | |  | | 174 (47) | |  | | |  | 44% | |  | | 47% | |  | |  |  |
| Pelvic lymph node dissection, n (%) | | |  | |  | |  | |  | |  | | |  |  | |  | |  | |  | |  |  |
| None | | |  | | 41 (50) | |  | | 194 (52) | | -0.067 | | |  | 55% | |  | | 51% | | -0.084 | |  |  |
| Limited | | |  | | 26 (31) | |  | | 97 (26) | |  | | |  | 27% | |  | | 27% | |  | |  |  |
| Extended | | |  | | 16 (19) | |  | | 82 (22) | |  | | |  | 18% | |  | | 22% | |  | |  |  |
| Neurovascular bundle preservation, n (%) | | |  | | 28 (34) | |  | | 108 (29) | | 0.103 | | |  | 26% | |  | | 30% | | -0.076 | |  |  |
| Preoperative hormonal therapy, n (%) | | |  | | 20 (24) | |  | | 100 (27) | | -0.062 | | |  | 30% | |  | | 26% | | 0.071 | |  |  |
| Perioperative continuation of antiplatelets, n (%) | | |  | | 12 (14) | |  | | 108 (29) | | -0.357 | | |  | 30% | |  | | 26% | | 0.081 | |  |  |
|  | | |  | |  | |  | |  | |  | | |  |  | |  | |  | |  | |  |  |
| ^ASA PS, American Society of Anesthesiologists physical status classification; BMI, body mass index; CKD, chronic kidney disease; eGFR, estimated glomerular filtration rate; IQR, interquartile range; KDIGO, Kidney Disease Improving Global Outcomes; NCCN, National Comprehensive Cancer Network; SD, Standardized difference^ | | | | | | | | | | | | | | | | | | | | | | |  |  |
|  |  |  |  |  |  |  |  |  |  |  |  |  |  |  |  |  |  |  |  |  |  |  |  | |

| (b) |  |  |  |  |  |  |  |  |  |  |  |  |  |
| --- | --- | --- | --- | --- | --- | --- | --- | --- | --- | --- | --- | --- | --- |
|  |  | ***Clopidogrel monotherapy group vs. Aspirin group (Ref.)*** | | | | | | | | | | |  |
| *Parameters* |  | ***Unweighted analysis*** | | | | |  | ***IPTW analysis*** | | | | |  |
| **Binary outcomes** |  | **Odds ratio** |  | **95% CI** |  | **P-value** |  | **Odds ratio** |  | **95% CI** |  | **P-value** |  |
| Bleeding complications |  | 3.73 |  | 0.98–14.19 |  | 0.054 |  | 6.63 |  | 2.78–15.86 |  | <0.001 |  |
| Low grade (C–D grade II or less) |  | 3.46 |  | 0.76–15.76 |  | 0.11 |  | 5.92 |  | 2.25–15.57 |  | <0.001 |  |
| High grade (C–D grade III or more) |  | 4.54 |  | 0.28–73.28 |  | 0.29 |  | 8.84 |  | 1.22–64.19 |  | 0.031 |  |
| Transfusion |  | 4.58 |  | 0.64–33.00 |  | 0.13 |  | 14.30 |  | 3.75–54.53 |  | <0.001 |  |
| Hemorrhagic shock |  | 4.54 |  | 0.28–73.28 |  | 0.29 |  | 8.84 |  | 1.22–64.19 |  | 0.031 |  |
| Thrombotic complication |  | NA |  | NA |  | NA |  | NA |  | NA |  | NA |  |
| Overall high-grade complications |  | 0.74 |  | 0.16–3.38 |  | 0.70 |  | 0.91 |  | 0.44–1.91 |  | 0.81 |  |
| Readmission |  | 2.30 |  | 0.56–9.37 |  | 0.250 |  | 2.22 |  | 0.93–5.30 |  | 0.072 |  |
|  |  |  |  |  |  |  |  |  |  |  |  |  |  |
| **Continuous outcomes** |  | **Estimate** |  | **95% CI** |  | **P-value** |  | **Estimate** |  | **95% CI** |  | **P-value** |  |
| Operation time, min |  | -2.42 |  | -12.18 to 7.34 |  | 0.63 |  | -4.64 |  | -12.40 to 3.12 |  | 0.24 |  |
| Estimated blood loss, mL |  | -9.08 |  | -37.60 to 19.43 |  | 0.53 |  | -54.28 |  | -90.46 to -18.09 |  | 0.003 |  |
| Hemoglobin deficit, median, mg/dL |  | -0.03 |  | -0.16 to 0.10 |  | 0.66 |  | -0.056 |  | -0.16 to 0.045 |  | 0.28 |  |
|  |  |  |  |  |  |  |  |  |  |  |  |  |  |
|  |  |  |  |  |  |  |  |  |  |  |  |  |  |
| ^C–D grade, Clavien–Dindo grade; CI, Confidence interval; IPTW, inverse probability of treatment weighting; NA, not available for zero event.^ | | | | | | | | | | | | |  |
|  |  |  |  |  |  |  |  |  |  |  |  |  |  |

| **Supplemental Table S4** Subgroup analysis: (a) Patient characteristics of subgroup analyses after IPTW compared between the thienopyridine subgroup and aspirin subgroup, in the cohorts of NCCN High-risk (left) and NCCN Low and Intermediate risk (right). (b) IPTW-regression models which analyzed associations between the outcomes and the group of the thienopyridine group compared on the aspirin group, in the cohorts of NCCN High-risk (left) and NCCN Low and Intermediate risk (right). | | | | | | | | | | |  |
| --- | --- | --- | --- | --- | --- | --- | --- | --- | --- | --- | --- |
| (a) |  |  |  |  |  |  |  |  |  |  |  |
|  |  | ***NCCN High-risk cohort*** | | | |  | ***NCCN Low and Intermediate risk cohort*** | | | |  |
| Parameter |  | Thienopyridine group | vs. | Aspirin group | SD (%) |  | Thienopyridine group | vs. | Aspirin group | SD (%) |  |
|  |  |  |  |  |  |  |  |  |  |  |  |
| Median age, years (IQR) |  | 72 (68-75) |  | 70 (67-74) | 0.028 |  | 69 (66-73) |  | 71 (68-74) | -0.019 |  |
| ASA PS, n (%) |  |  |  |  |  |  |  |  |  |  |  |
| 1 |  | 18% |  | 12% | 0.049 |  | 7.2% |  | 12% | 0.025 |  |
| 2 |  | 60% |  | 68% |  |  | 73% |  | 69% |  |  |
| 3, or more |  | 22% |  | 20% |  |  | 20% |  | 19% |  |  |
| BMI ≧ 25 kg/m2, n (%) |  | 38% |  | 40% | -0.041 |  | 31% |  | 37% | -0.127 |  |
| KDIGO CKD grade (eGFR), n (%) |  |  |  |  |  |  |  |  |  |  |  |
| Grade 1, or 2 (60 mL/min/1.73 m^2^, or more) |  | 67% |  | 66% | 0.021 |  | 61% |  | 62% | -0.021 |  |
| Grade 3a, or 3b (30–59 mL/min/1.73 m^2^) |  | 30% |  | 30% |  |  | 36% |  | 35% |  |  |
| Grade 4, or 5 (29 mL/min/1.73 m^2^, or less) |  | 3.0% |  | 3.4% |  |  | 2.6% |  | 2.6% |  |  |
| Pelvic lymph node dissection, n (%) |  |  |  |  |  |  |  |  |  |  |  |
| None |  | 32% |  | 28% | -0.022 |  | 72% |  | 74% | -0.089 |  |
| Limited |  | 37% |  | 40% |  |  | 20% |  | 15% |  |  |
| Extended |  | 31% |  | 32% |  |  | 7.5% |  | 11% |  |  |
| Neurovascular bundle preservation, n (%) |  | 20% |  | 20% | -0.013 |  | 42% |  | 39% | 0.061 |  |
| Preoperative hormonal therapy, n (%) |  | 43% |  | 42% | 0.020 |  | 10% |  | 11% | -0.033 |  |
| Perioperative continuation of antiplatelets, n (%) |  | 30% |  | 29% | 0.022 |  | 20% |  | 22% | -0.049 |  |
|  |  |  |  |  |  |  |  |  |  |  |  |
| ^ASA PS, American Society of Anesthesiologists physical status classification; BMI, body mass index; CKD, chronic kidney disease; IQR, interquartile range; NCCN, National Comprehensive Cancer Network; SD, Standardized difference^ | | | | | | | | | | |  |
|  |  |  |  |  |  |  |  |  |  |  |  |

| (b) |  |  |  |  |  |  |  |  |  |  |  |  | |  |
| --- | --- | --- | --- | --- | --- | --- | --- | --- | --- | --- | --- | --- | --- | --- |
|  |  | ***Thienopyridine vs. Aspirin subgroup (Ref.)*** | | | | | | | | | | | |  |
| *Parameters* |  | ***NCCN High-risk cohort*** | | | | |  | ***NCCN Low and Intermediate risk cohort*** | | | | | |  |
| **Binary outcomes** |  | **Odds ratio** |  | **95% C.I.** |  | **P-value** |  | **Odds ratio** |  | **95% C.I.** |  | **P-value** | |  |
| Bleeding complications |  | 4.66 |  | 1.43–15.22 |  | 0.011 |  | 3.40 |  | 1.05–11.01 |  | 0.041 | |  |
| Low grade (C-D grade II, or less) |  | 3.11 |  | 0.69–13.91 |  | 0.14 |  | 3.40 |  | 1.05–11.01 |  | 0.041 | |  |
| High grade (C-D grade III, or more) |  | 7.45 |  | 1.04–53.32 |  | 0.045 |  | NA |  | NA |  | NA | |  |
| Transfusion |  | 5.98 |  | 1.44–24.90 |  | 0.014 |  | NA |  | NA |  | NA | |  |
| Hemorrhagic shock |  | 7.45 |  | 1.04–53.33 |  | 0.045 |  | NA |  | NA |  | NA | |  |
| Thrombotic complication |  | 0.38 |  | 0.06–2.29 |  | 0.29 |  | NA |  | NA |  | NA | |  |
| Readmission |  | 3.94 |  | 1.50–10.36 |  | 0.005 |  | 2.09 |  | 0.46–9.57 |  | 0.340 | |  |
|  |  |  |  |  |  |  |  |  |  |  |  |  | |  |
|  |  |  |  |  |  |  |  |  |  |  |  |  | |  |
| ^C-D grade, Clavien-DIndo grade; C.I., Confidence interval; NCCN, National Comprehensive Cancer Network.^ | | | | | | | | | | | | | |  |
|  |  |  |  |  |  |  |  |  |  |  |  |  |  |  |
|  | | | | | | | | | | | | |  | |
